# Charcot–Marie–Tooth Disease With Episodic Rhabdomyolysis Due to Two Novel Mutations in the β Subunit of Mitochondrial Trifunctional Protein and Effective Response to Modified Diet Therapy

**DOI:** 10.3389/fneur.2021.694966

**Published:** 2021-10-12

**Authors:** Yuqing Guan, Yanxia Zhang, Xin-Ming Shen, Liang Zhou, Xuan Shang, Yu Peng, Yafang Hu, Wei Li

**Affiliations:** ^1^Department of Neurology, Nanfang Hospital, Southern Medical University, Guangzhou, China; ^2^Department of Medical Genetics, School of Basic Medical Sciences, Southern Medical University, Guangzhou, China; ^3^Department of Neurology, Mayo Clinic, Rochester, MN, United States

**Keywords:** mitochondrial trifunctional protein deficiency, HADHB, peripheral neuropathy, Charcot-Marie-Tooth disease, rhabdomyolysis

## Abstract

A 29-year-old female experienced chronic progressive peripheral neuropathy since childhood and was diagnosed with Charcot–Marie–Tooth disease (CMT) at age 15. She developed recurrent, fever-induced rhabdomyolysis (RM) at age 24. EMG studies showed decreased amplitude of compound muscle action potential, declined motor conductive velocity, and absence of sensor nerve action potential. Acylcarnitine analysis revealed elevated C16-OH, C18-OH, and C18:1-OH. Muscle biopsy showed scattered foci of necrotic myofibers invaded by macrophages, occasional regenerating fibers, and remarkable muscle fiber type grouping. Whole-exome sequencing identified two novel heterozygous mutations: c.490G>A (p.G164S) and c.686G>A (p.R229Q) in *HADHB* gene encoding the β-subunit of mitochondrial trifunctional protein (MTP). Reduction of long-chain fatty acid *via* dietary restrictions alleviated symptoms effectively. Our study indicates that the defect of the MTP β-subunit accounts for both CMT and RM in the same patient and expands the clinical spectrum of disorders caused by the HADHB mutations. Our systematic review of all MTPD patients with dietary treatment indicates that the effect of dietary treatment is related to the age of onset and the severity of symptoms.

## Introduction

Mitochondrial trifunctional protein (MTP) is a hetero-octamer complex consisting of four α subunits encoded by *HADHA* gene and four β subunits encoded by *HADHB* gene. It catalyzes three reactions in the fatty acid β-oxidation process by three enzymes located in the inner membrane of the mitochondria: long-chain enoyl-CoA hydrolase (LCEH) and long-chain 3-hydroxy-acyl-CoA dehydrogenase (LCHAD) contained in α subunits, and long-chain 3-ketoacyl-CoA thiolase (LCKT) contained in β subunits (1–4). Mitochondrial trifunctional protein deficiency (MTPD) is a rare autosomal recessive disorder caused by pathogenic variants of HADHA or HADHB. These variants disrupt MTP function with different severities resulting in complete MTP deficiency, isolated LCHAD or isolated LCKT deficiency (5). MTPD is clinically classified into three subtypes: (1) neonatal-onset type with cardiomyopathy and high mortality, (2) infant-onset type showing episodic hypoketotic hypoglycemia and hepatic dysfunction, and (3) later-onset neuromyopathic type with a wider spectrum of peripheral neuropathy, myopathy symptoms, or both (6). However, neither genotype–phenotype relation nor biochemistry–clinic correlation has been established due to its heterogeneities.

Charcot–Marie–Tooth (CMT) disease is an inherited peripheral motor and sensory neuropathy. It was traditionally classified into demyelinating, axonal, and intermediate subtypes based on nerve conduction velocity. A gene-based classification with mode of inheritance and neuropathy type was proposed (7). To date, more than 80 CMT-associated genes have been characterized (8–10). However, mutations in the β subunit of mitochondrial trifunctional protein (MTP) (*HADHB*) causing CMT have been detected in only six patients to date: compound heterozygous mutations in two CMT siblings with early onset axonal sensorimotor neuropathy (11), two unrelated infantile axonal CMT patients (12), and a homozygous mutation in two siblings with axonal CMT (13). All patients displayed typical motor sensory peripheral neuropathy without myopathic manifestation.

Rhabdomyolysis (RM) is characterized by skeletal muscle damage resulting in the release of intracellular muscle components such as myoglobin, creatine kinase (CK), aldolase, lactate dehydrogenase, and electrolytes into the bloodstream and extracellular space. RM symptoms can present from asymptomatic to renal failure depending on diverse environmental and genetic factors. Genetic defects associated with RM often account for various inherited neuromuscular and metabolic disorders including glycogen and fatty acid metabolism disorders, muscular dystrophies, and mitochondrial diseases (14). Although mitochondrial diseases usually prefer reduced ATP production due to primary downstream impairment of oxidative phosphorylation, additional essential metabolic processes such as beta-oxidation of long-chain fatty acids also occur within mitochondria. A total of 18 patients with RM caused by mutations in the *HADHB* gene were reported to date (patient 5–16, 24, 26, 29–32 in Table 1).

**Table 1:** Clinical manifestations and gene mutations in neuromuscular MTPD patients.

**Case No /Gender**	**Age at diagnosis**	**Evidence of myopathy/onset age**				**Evidence of PN/onset age**				**NCS/** **EMG**	**Gene**	**Mutation classification**	**cDNA**	**AA**	**Reference**
		**Prox W/MP**	**Exe Intol**	**RM**	**CK/max**	**Dist W/A**	**H/F deform**	**S abnorm**	**Refl-**						
Patients with both myopathy and neuropathy															
1/F	18 years	9 years	9 years	ND	>2xULN	9 years	9 years	Y	Y	PN	HADHA	com het	453 + 1G > T/955G > A	splicing defect/ Gly319Ser	(15)
2/M	?	Y?	Y	Y	Elevated	Y	Y	ND	ND	ND	HADHA	com het	871C>T/914T>A	Arg255ter/Ile269Asn	(16)
3/M	7 years	Y?	Y	Y	50,000	Y	Y	ND	ND	PN^*^	HADHA	homo	845T>A	Val246Asp	(16)
4/M	53 years	N	ND	48y	190,864	N	N	ND	Y	PN	HADHA	com het	1528G>C/180 + 3A>G	Glu510Gln/Thr37SerfsX6	(17)
5/M	13 years	ND	ND	Y	>10xULN	Y	ND	Y	Y	ND	HADHB	homo	712C > T	Arg238Trp	(15)
6/M	60 years	ND	childhood	55 years	40,000	40 years	40 years	40 years	Y	PN	HADHB	homo	1192T>C	Phe398Leu	(18)
7/F	20 years	before 20 years	<20y	20 years	193,936	childhood	childhood	childhood	Y	PN	HADHB	com het	209 + 1G>C/ 980T>C	splicing defect/ Leu327Leu	(19)
8/M	12 years	before 10 years	ND	10 years	26,000	before 10 years	4 years	ND	ND	PN	HADHB	homo?	341A>G	Asn114Ser	(20)
9/M	14 years	Y?	Y	Y	40,000	Y?	Y	ND	ND	PN	HADHB	com het	607C>T/881C>T	Arg170ter/Pro261Leu	(16)
10/M	4 years	Y?	Y	Y	ND	Y?	N	ND	ND	PN	HADHB	com het	607C>T/ 881C>T	Arg170ter/Pro261Leu	(16)
11/M	6 years	ND	Y	Y	60,000	ND	N	ND	ND	PN^*^	HADHB	homo	362T>C	Leu88Pro	(16)
12/M	18 years y	Y?	Y	Y	50,250	Y?	Y	ND	ND	PN^*^	HADHB	com het	176G>A/740G>A	Gly26Asp/Arg214His	(16)
13/F	29 years	7y	ND	23y	2656	Y	N	N	Y	ND	HADHB	com het	407T>C/421G>A	Met136Thr/Ala141Thr	(21)
14/F	8 years 5 month	birth	Y	likely	2735	Y	Y	ND	Y	PN	HADHB	homo	739C>T	Arg247Cys	(22)
15/F	18 years	N	ND	2 years	9,577	3 years	9 years	Y	Y	PN	HADHB	homo	1175C>T	Ala392Val	(23)
16/F	18 years	N	ND	3 years	ND	Y	Y	Y	Y	PN	HADHB	homo	1175C>T	Ala392Val	(23)
17/F	29 years	6 years	6 years	24 years	>10,000	Y	Y	Y	Y	PN	HADHB	com het	490G>A/686G>A	Gly164Ser/Arg229Gln	present case
Patients with neuropathy only															
18/M	19 years	N	ND	N	Normal	Y	Y	N	Y	PN	HADHB	com het	l 84A>G/ 340A>G	Thr62Ala/Asn114Asp	(12)
19/M	5 years	N	ND	N	400	Y	Y	N	Y	PN	HADHB	com het	488G>A/1175C>T	Gly163Asp/Ala392Val	(12)
20/M	34 years	N	N	N	ND	5 years	5 years	Y	5	PN	HADHB	com het	210-1G > C/686G > T	15aa deletion/ Arg229Leu	(11)
21/F	37 years	N	N	N	ND	6 years	ND	Y	Y	PN	HADHB	com het	210-1G > C/686G > T	15aa deletion/ Arg229Leu	(11)
22/F	29 years	N	ND	N	N	Childhood	Y	Y	Y	PN	HADHA	homo	955G > A	Gly319Ser	(13)
23/M	24 years	N	ND	N	N	Childhood	Y	Y	Y	PN	HADHA	homo	955G > A	Gly319Ser	(13)
Patients with myopathy only															
24/M	7 months	ND	Y	Y	165,000	ND	N	ND	ND	ND	HADHA	com het	1528G>C/ 1678C>T	Glu474Gln/ Arg524ter	(16)
25/M	17 years	4–5 years	4–5 years	ND	1.5xULN	ND	ND	ND	ND	ND	HADHA	com het^#^	180 + 3A > G/1528G > C	splicing defect/ Glu510Gln	(15)
26/F	12 years	ND	ND	4 years	37,935	ND	ND	ND	ND	ND	HADHB	com het	919A>G/1165A>G	Asn307Asp/Asn389Asp	(24)
27/F	12 years	Y?	ND	ND	1,000	Y?	ND	ND	ND	ND	HADHB	com het	182G>A/740G>A	Arg28His/Arg214His	(16)
28/M	10 months	Y?	N	ND	elevated	Y?	N	ND	ND	ND	HADHB	com het	181C>T/349A>G	Arg28Cys/Arg84Gly	(16)
29/F	8 years	Y?	Y	Y	29,960	Y?	N	ND	ND	ND	HADHB	homo	901G>A	Gly268Ser	(16)
30/M	3 years	Y?	Y	Y	elevated	Y?	N	ND	ND	ND	HADHB	com het	397A>C/881C>G	Thr100Pro/Pro261Arg	(16)
31/F	13 years	childhood	9 years	9 years	22,885	ND	ND	ND	ND	ND	HADHB	com het	520 C>T/1331 G>A	Arg141Cys/Arg411Lys	(25)
32/M	15 years	15 years	15 years	15 years	121,800	N	N	ND	ND	ND	HADHB	homo	1331G>A	Arg411Lys	(26)

*Case number 1 to 17: patients with both myopathy and peripheral neuropathy; Case number 18 to 23: patients with pure peripheral neuropathy; Case number 24 to 32: patients with pure myopathy*.

*AA: Amino acid changes; CK/max: CK elevation/maximal CK; com het: compound heterozygous; Dist W/A: distal weakness/atrophy; Elevated: elevated,but the precise number was not available; ESW: episodic severe weakness;Exe Intol: exercise intolerance; H/F deform: hand/feet deformity; homo: homozygous missense; MP: muscle pain; ND: not described; PN: peripheral neuropathy; Prox W: proximal weakness; Refl-: absent tendon reflexes; RM: rhabdomyolysis; S abnorm: sensory abnormality; ULN: upper limit of normal*.

# *Compound heterozygosity was not confirmed by analysis of parents*.

To date, seven patients with RM episodes and chronic peripheral neuropathy were characterized based on clinical manifestations and electrophysiological properties (patients 3, 6–8, 14–16 in Table 1). However, none were considered as CMT. Patients with various clinical manifestations including CMT or RM caused by HADHB mutations have been reported (22), but whether the *HADHB* mutation causes both CMT and RM in the same patient has not yet been determined. Moreover, early diagnosis of MTPD is now more easily achievable with newborn screening and next-generation sequencing (NGS), but studies on the treatment of MTPD have not increased proportionally. Here, we report a Chinese patient diagnosed with both CMT and RM caused by novel *HADHB* mutations with good response to dietary treatment.

## Methods

### Research Protocol and Consent Form

The study was approved by the Ethics Committee of Nanfang Hospital, Southern Medical University. The patient and the parents provided written informed consent for participation and publication of this study.

### Routine Examinations, Blood Acylcarnitine Analysis, and Urine Organic Acid Test

Routine blood examinations were implemented to evaluate levels of glucose, lipid, electrolytes, plasma lactic acid, hepatic and retinal function, serum creatine kinase (CK), myoglobin concentration, and parathyroid hormone. Chest X-ray, electrocardiogram, abdominal ultrasonography, and pulmonary ventilation test were also performed. We used gas chromatography-tandem mass spectrometry to assess blood acylcarnitine and urine organic acid levels.

### Nerve Conduction Study and Electromyography

Nerve conduction study and concentric needle electromyography (EMG) were performed with a Keypoint 9033A07 EMG machine (Dantec Corporation, Denmark). We also performed sympathetic skin response (SSR) and Ewing's test to assess the autonomic nervous system function.

### Skeletal Muscle Biopsy and Histochemical Staining

Biopsy of the left biceps, freeze fixation, frozen section, and staining of muscle specimen were performed according to the standard procedure in our laboratory (27).

### Genomic DNA Extraction and Whole-Exome Sequencing

EDTA-anticoagulated peripheral blood was collected, and genomic DNA was extracted. Whole-exome sequencing (WES) was performed using a standard PCR-based next-generation sequencing procedure on Illumina high throughput sequencer. The detected mutations were verified by Sanger sequencing. The pathogenicity of detected variations was predicted by SIFT, Polyphen-2, Mutation taster, PANTHER, and PROVEAN. I-Mutant 2.0 was used to assess the variant protein stability. The tertiary structure of the mutant protein was modeled by Swiss model software. Spatial structures of wild-type and mutant HADHB proteins were compared with PyMol.

## Results

### Clinical Information

A 29-year-old female patient was admitted due to childhood-onset progressive weakness and atrophy of distal limbs and intermittent fever-induced exacerbation of weakness for the past 5 years. She was born normally with normal movement development milestones in her early childhood. At the age of six, she started to feel tired after exercise and took frequent rest breaks during long walks. She was obviously inferior to her peers in sports performances. She gradually developed muscle atrophy in her hands and feet, weakness in legs, and abnormal gait. When she was 13 years old, she received heel tendon surgery, which temporarily improved her gait. At age 15, she was diagnosed with Charcot–Marie–Tooth disease in another neuromuscular disease clinic. She received physical therapy over the following years with little improvement. When she was 24 years old, she experienced marked exacerbation of weakness with severe general muscle pain after a fever induced by infection of upper respiratory tract. Thereafter, her strength deteriorated during each fever occurrence, causing her to remain bedbound for several days. Five months before her admission, she experienced her worst general weakness after developing a fever due to influenza. The patient had difficulty chewing and extending her neck, complained of severe pain in all extremities and trunk muscles, and noticed dark-colored urine. She was admitted to a local hospital and diagnosed with rhabdomyolysis with creatine kinase level >10,000 U/L. After hydration and nutritional support treatment for several days, she recovered to a pre-infection level. One month before her admission to our hospital, she underwent another infection-induced episode of exacerbation with similar symptoms and CK changes.

Physical examination showed normal cranial nerve signs. The muscular volumes in her upper arms, forearms, and proximal thigh were normal. Mild muscle atrophy was found in both hands with grip strength of 4/5 (Medical Research Council grade scale). She had muscle atrophy in bilateral distal thighs and legs with high-arched feet. Muscle strength was 2/5 in foot dorsal extension and 4/5 in hip flexion, knee extension, knee flexion, and foot plantar flexion. Pain sensation of upper limbs was normal, whereas vibration sensation below the elbow was decreased, and pain and vibration sensation below both knees were diminished. Tendon reflexes in all extremities were absent. She had been wheelchair bound for the last 2 years and needed assistance to rise from the wheelchair. She could stand on her own but had difficulty walking, with a mixture of stoppage and waddling gait. Ophthalmic examination showed normal findings. Her parents were asymptomatic with normal muscle strength, tendon reflex, and sensation.

### Routine Blood Test and Other Examinations

Both serum CK and myoglobin levels were elevated to 450 and 336 IU/L from normal ranges of 2–178 and 0–110 IU/L, respectively. The plasma lactic acid was mildly increased to 2.9 mmol/L from a normal range of 0–2.2 mmol/L. Abdominal ultrasound showed fatty liver. Serum calcium, phosphate, parathyroid hormone, and other routine tests were normal. Head CT scan showed no abnormality.

### Electromyography Studies

Motor nerve conductions were measured in bilateral median, ulnar, tibial, and peroneus communis nerves. Compound muscle action potential (CMAP) and motor conductive velocity (MCV) were both reduced in all tested nerves. The CMAP of upper extremities ranged from 4.0 to 5.3 mV, whereas the CMAP of lower extremities ranged from 0.47 to 2.3 mV. The MCV of upper extremities ranged from 26.5 to 46.0 m/s, whereas the MCV of lower extremities ranged from 29.2 to 38.5 m/s. The F-wave rate of the right tibial nerve decreased to 20% of control with normal latency. Sensor nerve conductance was measured in bilateral median, ulnar, peroneus communis, and sural nerves. No sensory nerve action potential (SNAP) was recorded in any of the tested nerves. Electromyography studies were performed in the right deltoid, extensor digitorum communis, vastus medialis, and tibial anterior muscles. All showed moderate fibrillation and positive sharp waves with reduced interference pattern. Compared with control with the same age group, the duration of motor unit potential (MUP) was prolonged from 18% to 67%. Sympathetic skin response and Ewing's test showed normal function of unmyelinated nerve fibers.

### Metabolic Tests

Acylcarnitine analysis of blood spot obtained during the interval of rhabdomyolysis attacks revealed mild elevation of C16-OH (0.18 *μ*mol/L vs. normal range 0.01–0.10 *μ*mol/L), C18-OH (0.07 *μ*mol/L vs. normal range 0.00–0.05 *μ*mol/L), and C18:1-OH (0.15 *μ*mol/L vs. normal range 0.01–0.10 *μ*mol/L). Urine organic acid level was normal. The results indicate a biochemical phenotype of an MTP multienzyme complex deficiency.

### Muscle Biopsy

**Figure 1:**
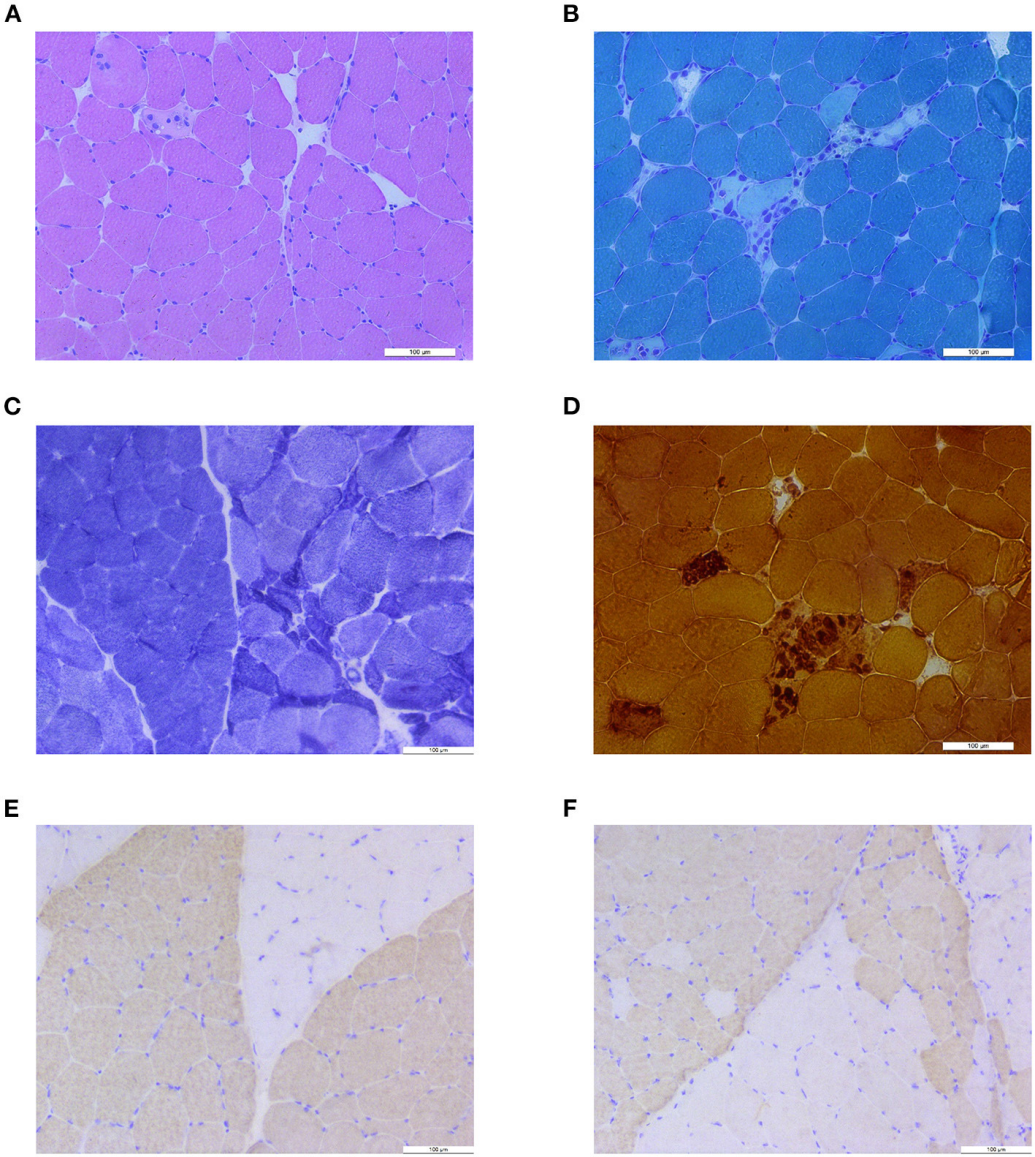
Muscle biopsy results. **(A)** H&E stain showed isolated necrotic muscle fiber and atrophic muscle fibers. **(B)** Modified Gomori trichrome (MGT) stain showed small cluster of necrosis and macrophage of muscle fibers. **(C)** Nicotinamide adenine dinucleotide tetrazolium oxidoreductase (NADH-TR) stain revealed grouping of myofibers and a small group of atrophic fibers. **(D)** Nonspecific esterase (NSE) staining showed necrosis and phagocytosis of muscle fibers. **(E)** Anti-fast-myosin immunohistochemistry (IHC) stain showed grouping of type 1 (pale) and type 2 (dark) myofibers. **(F)** Anti-slow-myosin IHC stain showed grouping of type 1 (dark) and type 2 (pale) myofibers.

Biopsy of the left biceps revealed scattered foci of necrotic myofibers invaded by macrophages and occasional regenerating fibers, which were consistent with the episode of rhabdomyolysis (Figures 1A,B,D). No pathological changes suggested abnormal lipid metabolism, abnormal glucose metabolism, or abnormal mitochondria. A small cluster of atrophic myofibers was found (Figure 1C). No target fiber was investigated. Remarkable muscle fiber type grouping appeared on NADH-TR stain and on fast-myosin and slow-myosin immunohistochemistry (IHC) stain, indicating a long-standing process of reinnervation, which was consistent with the clinical procedure of CMT (Figures 1C,E,F).

### Genetic Analysis

**Figure 2:**
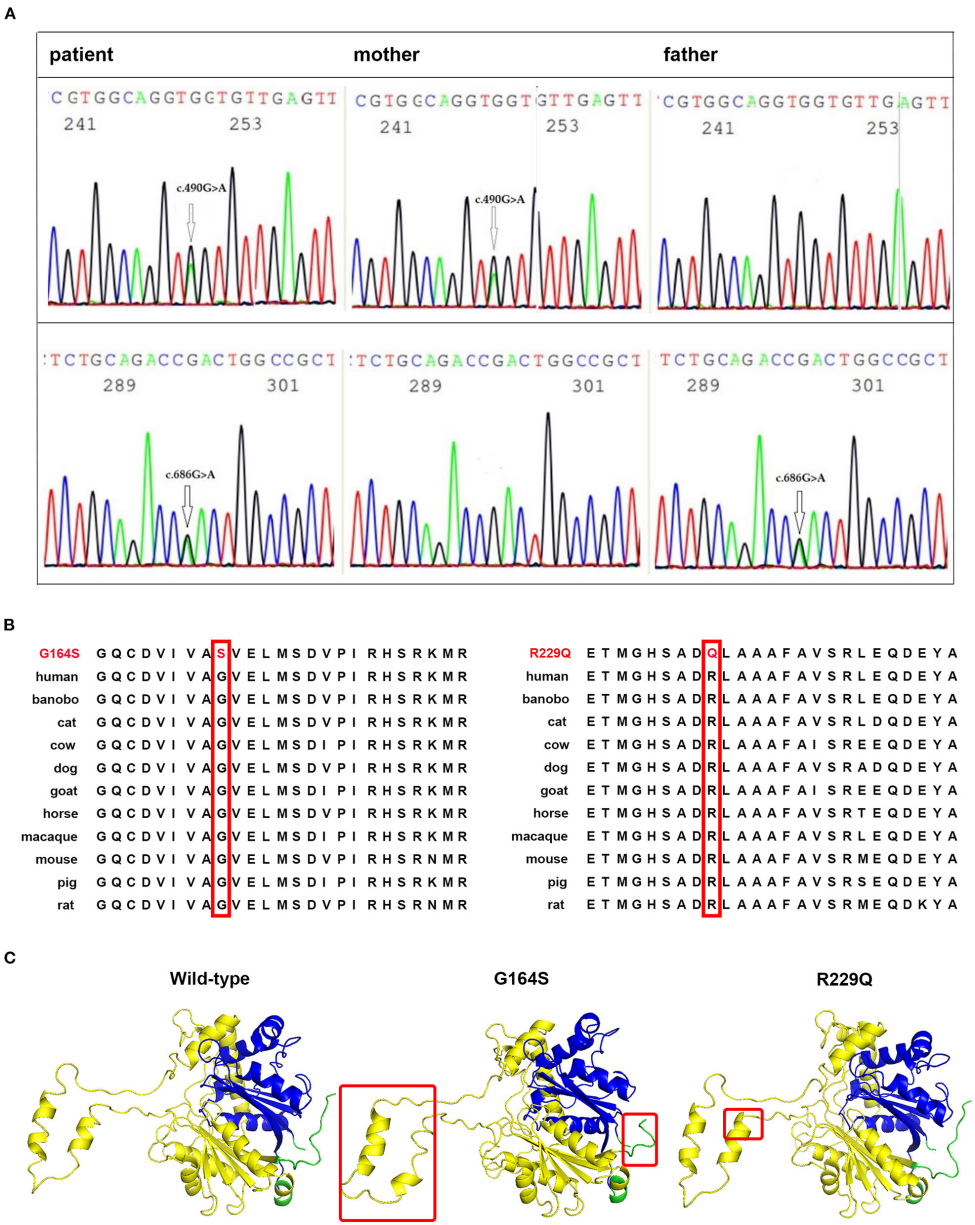
Identification of disease-associated HADHB mutations. **(A)** Sanger sequencing showing the compound heterozygous mutations, c.490G>A and c.686G>A, in the *HADHB* gene of the patient and heterozygous mutation in her parents. **(B)** The glycine residue at codon 164 and arginine residue at codon 229 of *HADHB* protein are highly conserved among different species (shown in red boxes). **(C)** Comparison of spatial structure of the wild and mutant *HADHB* proteins as indicated. The *HADHB* protein has two domains where the gene encodes thiolase enzyme, N-terminal domain (yellow chain), and C-terminal domain (blue chain). Modified points are marked with a red rectangle.

Whole-exome and Sanger sequencing identified two heterozygous variants in *HADHB* gene (NM_000183.2) encoding MTP β subunit: c.490G>A (p.G164S) in the paternal allele and c.686G>A (p.R229Q) in the maternal allele (Figure 2A). The c.490G>A was not found in variance database or literature, whereas c.686G>A variant has been listed in the ClinVar database (variation ID: 253051) with uncertain clinical significance. Both variants were predicted to be damaged mutation by Mutation Taster, PROVEAN, Polyphen-2, and PANTHER analysis, and to decrease the stability of the protein structure by I-Mutant analysis. Both mutations are located in the N-terminal domain of HADHB protein, and mutant residues are conserved in all species (Figure 2B). Structure modeling showed that compared with wild-type protein, a part of the α-helix in N-terminal domain of G164S protein changes into a strand, and a part of strand in N-terminal domain of R229Q protein turns into a helix (Figure 2C). The tail structure in G164S protein was also altered.

### Treatment

The patient was given a low-fat, high-carbohydrate diet. She ate cereals (including rice, corn, brown rice, flour, and sorghum), bean products (other than soybeans), pig blood, chicken blood, vegetables, and fruits without controlling the total amount. She also consumed a total of 50 g of steamed sea fish, shrimp, and peeled poultry meat daily. She drank 500 ml of skimmed milk every day and consumed one egg white and 10 g of nuts as well. She supplemented with 35 g of medium-chain triglyceride and 30 g of whey protein powder, which were divided into three doses daily. The daily fat intake was limited to 43 g, including 36 g of medium-chain fat and 7 g of long-chain essential fat. She also had 100 mg of L-carnitine with multivitamin tablet daily to prevent deficiency of lipid soluble vitamins. She avoided cooking oil and prolonged fasting and exercise. The motor function of the patient gradually improved and reached a plateau after 1 year of the diet treatment. After that, she stopped taking medium-chain triglyceride powder because of its poor taste. She still follows the low-fat, high-carbohydrate diet principle. In a recent follow-up, ~2.5 years after the diet treatment, neurological examination showed that muscle strength was 4/5 in grip, 2/5 in foot dorsal extension, and 4/5 in hip flexion, which were comparable with initial measurement. However, the power of knee extension, knee flexion, and foot plantar flexion improved to 5/5. Examination of muscle volume, sensation, and tendon reflexes showed results similar to the start of the study. The result of a 6-min walking test was 225 m. The motor function of the patient improved greatly compared with pretreatment levels, when she could barely walk and required another person to carry her up and down the stairs. Now, she can walk up to the third floor without taking a break. She did not experience RM again, even during the process of respiratory tract infection and fever, which happened 6 months after the start of dietary treatment. The patient works as a volunteer in a nursing home, helping elderly individuals with documents and computer-related matters.

## Discussion

Based on the clinical symptoms, electrophysiological findings, acylcarnitine analysis results, and pathologic changes, and episodic fever-triggered RM at a later age, our patient was diagnosed with MTPD with manifestation of childhood-onset CMT and paroxysmal RM after adulthood and showed good response to dietary therapy. We traced the CMT and RM of this patient into two novel mutations G164S and R229Q in *HADHB* with each likely altering the structure of the protein. To our knowledge, this is the first report of CMT and RM caused by *HADHB* mutations in the Chinese population.

To date, 32 total patients with neuromyopathic MTPD were reported with 8 harboring *HADHA* mutations and 24 carrying *HADHB* mutations (Table 1). Of 32 patients, six displayed peripheral neuropathy, nine with myopathy, and 17 with both peripheral neuropathy and myopathy (Table 1). We classify the clinical manifestations of neuromuscular MTPD into three categories: (1) peripheral neuropathy with symptoms and electrophysiological changes of CMT—usually more severe in lower limbs, (2) myopathy presented as episodic rhabdomyolysis triggered by stressful situations and with milder myopathic symptoms such as exercise intolerance, proximal weakness, and muscle pain, and (3) other complications, such as pigmentary retinopathy and hypoparathyroidism. The neuromuscular (NM) subtype of MTPD was previously defined as mild or adult-onset (22, 28), but that is not always the case. Although the age of onset in some MTPD patients is as late as 48 years of age (17), the first episode of rhabdomyolysis may occur within the first year of life with life-threatening symptoms (16). Some patients survived from infant hepatopathy but developed PN in later life (20). MTPD patients with NM defects demonstrated a very wide spectrum of clinical manifestations. The features of PN can be subtle or even subclinical in some patients (17). On the other hand, in patients with obvious peripheral neuropathy symptoms, myopathic features such as mild proximal weakness and exercise intolerance might be overlooked. That was the case with our patient. With detailed inquiry, she recalled fatigue after exercise since childhood and general fatigue during her menstrual period. Our systematic review on all reported patients with mutations in *HADHA* and *HADHB* suggests there is no correlation between genotype and phenotype.

Early-onset axonal CMT caused by heterozygous mutations in *HADHB* was first described in two Korean siblings in 2013 (11). Infant- and childhood-onset CMT caused by *HADHB* mutations were characterized in two unrelated Chinese patients. Recently, a homozygous missense *HADHA* mutation was reported in two Iranian siblings with clinical manifestation of axonal form of CMT (CMT2) (12, 13). Recently, it was suggested that peripheral neuropathy related to HADHB mutations may be manifested as axonal CMT without other MTP deficiency symptoms (29). However, the diagnosis of CMT in patients with MTPD was considered “misdiagnosed” in another report (6). The present study supports the notion that *HADHA*- or *HADHB*-related motor and sensory neuropathy should be considered as a CMT subtype. Our patient was diagnosed with axonal CMT at the age of 15 based on her clinical course and electrophysiological changes without identification of pathogenic mutation. If the *HADHB* gene had been included in the screening panel for hereditary peripheral neuropathy, our patient would have received the correct genetic diagnosis 14 years earlier, resulting in proper treatment with better outcomes and improved quality of life. Her rhabdomyolysis episodes could have been avoided. Thus, we report a new subtype of CMT with metabolic myopathy due to MTPD deficiency caused by mutations of *HADHB* gene, suggesting that all CMT patients without definite pathogenic mutations should be screened for mutation in *HADHA* and *HADHB* genes.

Prognosis of MTPD may be significantly improved by diet-modulating therapy with appropriate prevention of energy decompensation (20). Dietary modification and avoidance of stressful triggers are particularly effective in preventing attacks of rhabdomyolysis (31). Guidelines on treatment of MTPD are not yet agreed upon and established to date. The current recommendation on dietary modification includes carbohydrate-based diet with supplementation of medium-chain triglycerides and restriction of long-chain fatty acid (5, 32). We reviewed literatures on dietary treatment of all MTPD patients. Eighteen patients with detailed descriptions of therapeutic strategies and clinical outcomes are summarized in Table 2. We found that the effect of dietary treatment was related to the age of onset and the severity of symptoms. All four MTPD patients of neonatal-onset subtype died, even after timely treatment, with a maximum life span of only 9 months. In six patients with infant-onset hypoglycemia and/or hepatopathy, two died at nine and 10 months, respectively, and four had favorable outcomes. Five patients with NM subtype had positive reactions to dietary treatment. It is noteworthy that three patients (patients 11, 13, and 17 in Table 2) with early onset and severe symptoms improved explicitly after treatment. These patients were diagnosed and treated at age 14 years (patient 11), 7 years (patient 13), and 10 years (patient 17), respectively. The mutations were in HADHA for patient 11 and HADHB for patients 13 and 17. In these patients, more treatment points (at least five out of 10) were implemented. The dietary treatment of our case, which started at the age of 29 years, also achieved satisfactory results, suggesting that MTPD is more treatable than expected. However, in the literature, late-onset neuromuscular MTPD with outcome of dietary treatment only accounted for a small portion of this subtype. A possible reason is that adult patients showed poor compliance to the recommended dietary plan (5, 19). Our patient reacted well because we tailored a diet regimen in accordance with the above principles by combining formula nutrients with selected foods, which was more readily accepted and adhered to by the patient. The notable improvement in her gait and exercise endurance enhanced her compliance to the diet. Therefore, our modified strategy of diet treatment can be applied to late-onset MTPD patients who have difficulties adhering to stricter diet modification.

**Table 2:** Dietary treatment of MTPD patients.

**No**	**Age**	**Gender**	**Onset age**	**Symptom summary**	**Subtype**	**Gene**	**Mutation**	**Treatment**	**Outcome**	**Reference**
1^*^	48 days	F	newborn	Hyperammonemia hyperlactatemia	Neonatal	HADHA	homo c.1689 + 2 T>G#	2,3,4,6	died of respiratory arrest	(24)
2^*^	26 days	F	newborn	Uncontrollable acidosis hypoglycemia; cardiac failure	neonatal	HADHA	homo c.1689 + 2 T>G#	2,3,4,6	died of multi-organ failure	(24)
3	21/2 weeks	F	1 weeks	hypoglycemia; metabolic crisis; hepatopathy; hypotonia; cardiomyopathy; seizures	neonatal	HADHA	homo c.1528G>C	4,6	died of cardiorespiratory failure	(30)
4	9 months	F	neonatal	urinary protein +; dicarboxyl aciduria; failure to thrive; hypoglycemia hepatopathy; hypotonia; cardiomyopathy; retinopathy	neonatal	HADHA	homo c.1528G>C	2,4	died of respiratory failure	(30)
5	14 years	F	<5 months	Hepatomegaly; hypoglycemia episodes of RM; cardiomyopathy retinopathy; generalized seizures; acute hepatic failure; cardiac failure	infant	HADHA	hetero c.1528G>C	◇,8	episodes of RM resolved; acute hepatic and cardiac failure stabilized; significant learning difficulties at age 7; PN and PR identified at age 14	(20)
6	14 years	F	1 year 5 months	Hypoglycemia; hepatopathy hypotonia; cardiomyopathy retinopathy; seizures neuropathy; retardation	infant	HADHA	homo c.1528G>C	4,6	hypoglycemia and acute deteriorations subsided; heart and liver no longer enlarged; CK intermittently elevated without symptoms of RM; occasional tonic-clonic seizures;	(30)
7	9 months	F	4 months	Hypoglycemia; metabolic crisis hepatopathy; hypotonia cardiomyopathy; retinopathy; seizures; retardation	infant	HADHA	homo c.1528G>C	2,4	died of pneumonia, cardiorespiratory failure	(30)
8	10 months	M	6–10 months	motor retardation; metabolic crisis hypoglycemia; hepatopathy hypotonia; cardiomyopathy retinopathy; seizures; retardation	infant	HADHA	homo c.1528G>C	2.4	died of cardiorespiratory failure	(30)
9	10 years	M	4 months	Hypoglycemia lethargy episodic severe weakness	infant	HADHA	c.1528G>C c.1678C>T	1,2,4,6,7,8	episodes of RM occurred less frequently; pigmentary retinopathy did not progress after the implementation of DHA; no visual impairment at age 10	(16)
10	15 years	F	5 months	Hypotonia; hepatomegaly hypoketotic hypoglycemia; hypertrophic cardiomyopathy pigmentary retinopathy low DHA levels	infant	HADHA	homo c.1528G>C	1,2,4, 8	cardiomyopathy fully resolved at 5y; progressive pigmentary retinopathy; stabilization of visual function after DHA supplementation	(20)
11	19 years	M	20 months	progressive weakness, episodic severe weakness	NM	HADHA	homo c.845T>A	1,2,3,4,67,8	episodes of RM occurred less frequently; reversal of peripheral sensorimotor neuropathy clinically and by NCS	(16)
12	53 years	M	48 years	recurrent RM	NM	HADHA	c.1528G>C c. A180 + 3>G	1,2,3,5	no additional episodes of RM	(17)
13	13 years	M	15 months	gait abnormalities; Achilles tendon tightness; PN; progression of weakness (needed wheelchair at age 10); episodes of RM; extreme fatigability; pigmentary retinopathy	NM	HADHB	c.341A>G	1,4,7,9 8 (age 12)	marked improvement in mobility and general strength; NCS showed improvement	(20)
16	20 years	F	40 days	shuddering attacks with reduced consciousness; hypocalcemia hypoparathyroidism; child-onset axonal motor sensory polyneuropathy; muscle pain and cramps after exercise; RM preceded by fever, vomiting, and diarrhea	NM	HADHB	c.209 + 1G>C c.980T>C	2,3,4,9,10	exercise endurance improved	(19)
17	14 years	M	1–2 years	severely disabling neuropathy; episode of RM started at 13.5 years of age	NM	HADHB	c.607C>T, c.881C>T	1,2,3,7,8	weakness significantly improved	(16)
18	29 years	F	6 years	child-onset progressive PN; episodic RM started at age 24	NM	HADHB	c.490G>A, c.686G>A	1,2,3,4, 5,6,7	no more episode of RM; weakness and exercise endurance improved	present

* *monozygotic twins*;

# *the mutation lead to the skipping of exon 16 with an in-frame 69-bp deletion*;

◇ *short-term dietary treatment (not described in detail). NM, neuromuscular subtype; NCS, nerve conduction studies; PN, peripheral neuropathy; RM, rhabdomyolysis. Treatment points: 1: avoidance of prolonged fasting; 2: restriction of long-chain fatty acids; 3: supplementation of medium-chain triglyceride oil; 4: low-fat high-carbohydrate diet; 5: daily multivitamin; 6: L-carnitine supplementation; 7: avoidance of prolonged exercise; 8: docosahexanoic acid (DHA) supplementation; 9: cornstarch supplementation at night; 10: maltodextrin*.

## Conclusion

We identified two novel variants in HADHB, G164S and R229Q, causing CMT and RM. The *HADHA* and *HADHB* gene should be added into the screening panel for CMT and metabolic myopathy. An MTPD patient in the neuromuscular subtype demonstrated significant improvement from a diet with a combination of selected food, supplemental medium chain triglyceride, and other nutrition.

## Data Availability Statement

The datasets presented in this article are not readily available due to ethical and privacy restrictions. Requests to access the datasets should be directed to the corresponding author.

## Ethics Statement

The studies involving human participants were reviewed and approved by Medical Ethics Committee of NanFang Hospital of Southern Medical University. The patients/participants provided their written informed consent to participate in this study. Written informed consent was obtained from the individual(s) for the publication of any potentially identifiable images or data included in this article.

## Author Contributions

YG, YZ, and X-MS conducted the material preparation, data collection, and analysis. YG wrote the first draft of the manuscript. All authors commented on previous versions of manuscript, contributed to the study conception, design, read and approved the final manuscript.

## Funding

This work was supported by the Guangdong Basic and Applied Basic Research Foundation (2019A1515011545) and Southern Medical University Innovation Training Program for Undergraduate Students (201912121027).

## Conflict of Interest

The authors declare that the research was conducted in the absence of any commercial or financial relationships that could be construed as a potential conflict of interest.

## Publisher's Note

All claims expressed in this article are solely those of the authors and do not necessarily represent those of their affiliated organizations, or those of the publisher, the editors and the reviewers. Any product that may be evaluated in this article, or claim that may be made by its manufacturer, is not guaranteed or endorsed by the publisher.
